# Nanoparticle-Based Modulation and Monitoring of Antigen-Presenting Cells in Organ Transplantation

**DOI:** 10.3389/fimmu.2017.01888

**Published:** 2017-12-22

**Authors:** Jordi Ochando, Mounia S. Braza

**Affiliations:** ^1^Department of Oncological Sciences, Icahn School of Medicine at Mount Sinai, Immunology Institute, New York, NY, United States

**Keywords:** nanoparticles, innate immune system, transplantation immunology, tolerance, therapeutics

## Abstract

Donor-specific unresponsiveness while preserving an intact immune function remains difficult to achieve in organ transplantation. Induction of tolerance requires a fine modulation of the interconnected innate and adaptive immune systems. Antigen-presenting cells (APCs) predominate during allograft rejection and create a highly inflammatory context where allospecific T cells are primed. Currently, the available protocols to prevent allograft rejection include a cocktail of drugs that are efficient in the short-term, but with severe long-term side effects and considerable toxicity. Consequently, better and less burdensome strategies are needed to promote indefinite allograft survival. Targeted delivery of immunosuppressive drugs that prevent the alloimmune response may address some of these problems. Nanoparticle-based approaches represent a promising strategy to negatively modulate the alloresponse by specifically delivering small compounds to APCs *in vivo*. Nanoparticles are also used as integrating imaging moieties to monitor inflammation for diagnostic purposes. Therefore, nanotechnology approaches represent an attractive strategy to deliver and monitor the efficacy of immunosuppressive therapy in organ transplantation with the potential to improve the clinical treatment of transplant patients.

## Introduction

Transplantation is a life-enhancing therapeutic option for tens of thousands of patients with end-stage organ failure. Outstanding short-term outcomes in organ transplantation have been achieved by pharmacologic immunosuppression. Despite these accomplishments, the detrimental effects’ life-long continuous immunosuppression compromise long-term allograft survival (1, 2). Immunosuppressive combination therapies are not specific and often toxic, resulting in the deterioration of the patient quality of life and severe side effects, including infections and malignancies (3, 4).

Novel therapeutic approaches that target the adaptive immune response have been developed, but the long-term transplant outcomes remain suboptimal. This underlines the need for additional approaches to develop tolerance-inducing protocols. Allograft tolerance induction in murine models cannot be fully explained by mechanisms that target only the adaptive immunity (5, 6). Recent work revealed how the innate immune system, especially monocytes and macrophages, reacts to allogeneic non-self and critically influences the adaptive immune response (7–10). As a result, therapeutic approaches that target myeloid cells *in vivo* and deliver immunomodulatory agents that prevent activation of the adaptive immune response represents a largely unexplored approach to promote indefinite allograft survival.

In this mini review, we first discuss the current state and perspectives of nanotherapy in transplantation by focusing on nanoparticles, particularly for modulation and immunosuppressive drug delivery to antigen-presenting cells (APCs). We then introduce the synthetic high-density lipoprotein (HDL) nanoparticles (HDL-NPs), which represent an emerging and very promising nanotherapeutic option to be exploited in organ transplantation. In addition, we describe nanoparticle-based imaging approaches that are being evaluated for graft immune monitoring and transplant rejection diagnosis. We finally raise several outstanding questions about the use of nanoparticles in organ transplantation to conclude that this technology represents an additional therapeutic option to prevent transplant rejection and promote organ acceptance.

## APCs as a Therapeutic Target for Immunosuppressive Therapy in Transplantation

Circulating and tissue-specific monocytes, macrophages, and dendritic cells (DCs) are APCs that activate strong cellular and humoral immune response against the transplanted organ. Non-self recognition by the innate immune system is certainly required for this response; however, it is still unclear what other mechanisms are involved in the early steps leading to APC maturation. It has been hypothesized that dying graft cells release “danger” molecules that directly induce APC maturation and that then initiate the adaptive alloimmune (11). Fadi Lakkis laboratory demonstrated that the “danger” signal associated with dying cells is not sufficient to initiate alloimmune response but that innate recognition of allogeneic non-self is required (9). By analyzing the innate immune response in either syngeneic or allogeneic grafts, it was demonstrated that only allogeneic grafts induced persistent differentiation of recipient monocytes into mature DCs that expressed interleukin 12 (IL-12) and stimulated T-cell proliferation and interferon γ (IFN-γ) production (9, 11). Altogether, these findings underline the importance of alloantigen innate recognition by APCs in initiating graft rejection and in maintaining a pro-inflammatory context. More recently, the Lakkis laboratory uncovered the mechanisms underlying non-self allorecognition and demonstrated that donor polymorphism in the gene encoding the signal regulatory protein α recognition by recipient CD47 elicits the innate immune response (12).

While monocyte-derived cell accumulation in transplanted organs has long been recognized as a feature of allograft rejection (13), recent data suggest that monocyte-derived macrophages inhibit graft-reactive immune responses (14) and mediate the induction of transplantation tolerance (10). This suggests that the functional properties (stimulatory or suppressive) of allograft-infiltrating APCs dictate the outcome of the transplanted organ. In this respect, circulating stimulatory (Ly-6C^hi^) monocytes contribute to leukocyte recruitment and consequently to acute organ rejection (15), while suppressive (Ly-6C^lo^) macrophages are responsible for the long-term allograft survival (10). These findings indicate that the innate immune system is not just an innocent bystander in the allograft immune response and that its modulation is required for tilting the immune balance in favor of the homeostasis status and of long-term allograft survival.

## Nanoparticle-Based Modulation of APCs for Transplantation Tolerance

**Figure 1:**
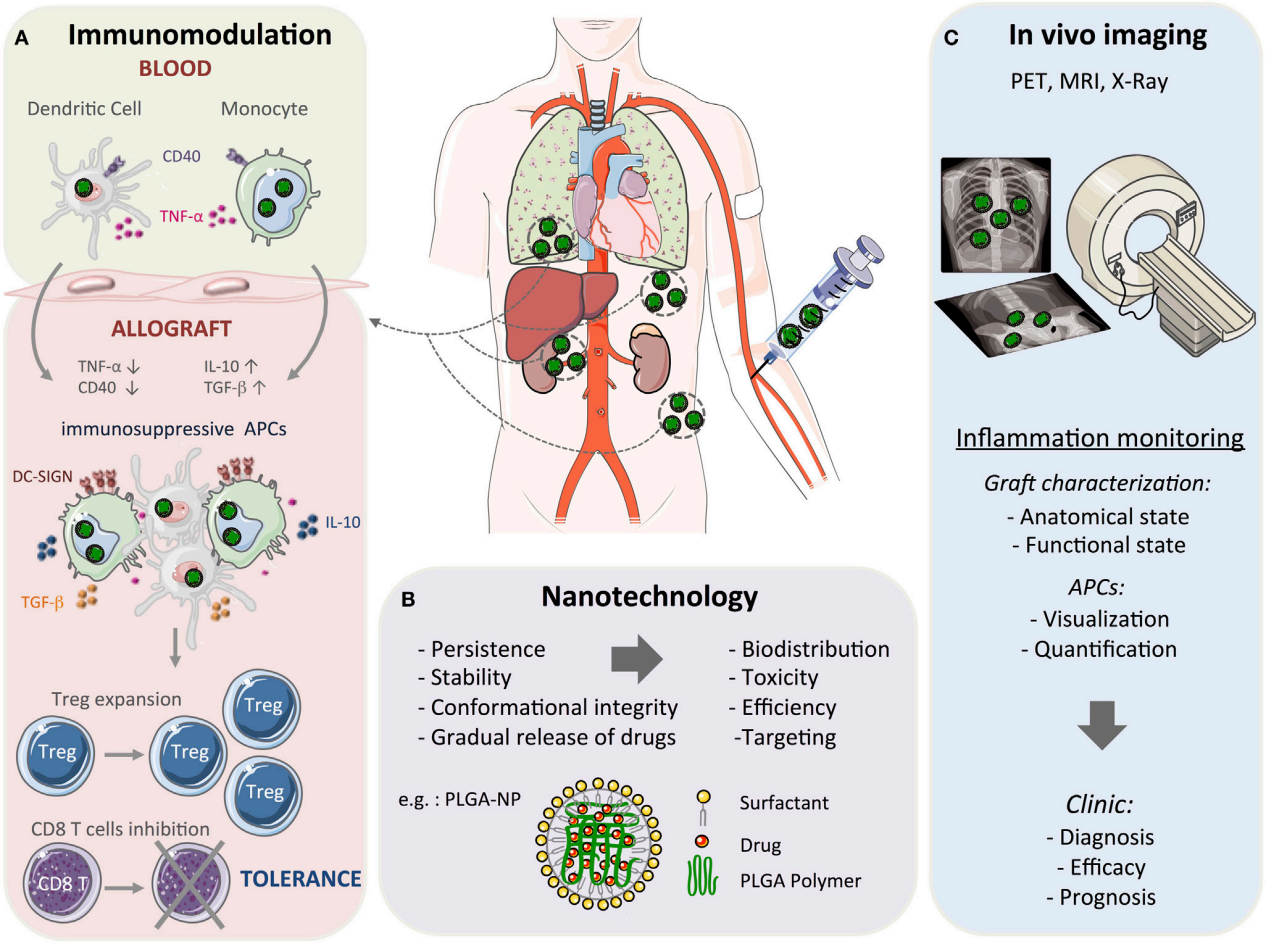
Toward nanomedicine in transplantation. Conventional organ transplant treatment requires continuous immunosuppressive drugs to provide therapeutic benefit that results in several side effects, including toxicity. Due to their structural stability and gradual drug release capacity, nanoparticle-based strategies could be used to reduce drug doses, minimize toxicity, and induce long-term allograft tolerance. Among the nanomaterials currently being developed, many are studied as drug delivery and imaging agents. **(A)** Myeloid cells can be targeted by using nanoparticles (in green) with the aim of modulating the early steps of the immune response. Nanoparticles deliver immunosuppressive drugs and/or antigens that result in a tolerogenic environment through the upregulation of anti-inflammatory mediators, such as IL-10, TGF-β, and the Dendritic Cell-Specific Intercellular adhesion molecule-3-Grabbing Non-integrin (DC-SIGN), and the downregulation of pro-inflammatory mediators, such as TNF-α and CD40. This will promote the formation and maintenance of myeloid cells with suppressive activity to reduce the alloreactive T cell response and concomitantly induce regulatory T cells (Treg) and long-term tolerance. **(B)** Among the various polymers synthesized for formulating polymeric nanoparticles, poly(lactic-co-glycolic acid) (PLGA) is the most popular with several interesting properties such as controlled and sustained release, low cytotoxicity, biocompatibility with tissues and cells, and a targeted delivery. A schematic representation of PLGA-based nanoparticles (PLGA-NP) is included in this figure. The entrapped drug is distributed throughout the polymer matrix and the particles surface is covered with a cationic surfactant such as didodecyldimethylammonium bromide. **(C)** Nanoparticles can also be used as moieties for positron emission tomography (PET), magnetic resonance imaging (MRI), and X-ray imaging and monitor graft function in patients. These non-invasive imaging approaches could be applied for diagnosis and prognostic purposes.

Drug-loaded nanoparticles represent a promising tool in organ transplantation to circumvent the limitations of conventional approaches by a localized, sustained, and controlled delivery of bioactive agents. Engineering nanoparticles for modulating the innate immune system in transplantation is an emerging field that provides new insights into the basic immunobiology of graft rejection/tolerance. The therapeutic aim is to deliver antigens and immune modulatory agents through specific myeloid derived cell targeting, thus allowing a better control on the innate immune response to induce transplantation tolerance (Figure 1A).

Targeting DCs with nanoparticles harboring antibodies or small compounds is one of the most promising strategies to negatively regulate the immune response after transplantation. Delivering antigen to specific DC receptors may result in the production of regulatory cytokines and the induction of negative costimulatory pathways that promote tolerogenic responses. C-type lectin receptors that are responsible of antigen presentation, such as mannose receptor and DEC-205 (16), have been previously used for immune cell activation (17, 18). Interestingly, antigen delivery by the same nanoparticles in the absence of adjuvant induces suppressive immune responses, leading to a tolerogenic phenotype (19). This represents a potential strategy to inhibit activated CD4 and CD8 T cells that mediate transplant rejection. Furthermore, transplant recipient mice treated with nanoparticle-encapsulated immunosuppressive drugs, such as rapamycin, tacrolimus, and mycophenolic acid, prolong allograft survival. PLGA nanoparticles have been developed to deliver rapamycin to increase the suppressive activity of myeloid cells. The resulting nanoparticles have a better efficacy in comparison to free drug in terms of antiproliferative (20), and inhibitory effects on the maturation of DCs (21). In a mouse model of skin transplantation, Goldstein and colleagues successfully delivered mycophenolic acid loaded-PLGA nanoparticles to myeloid cells, which prolonged allograft associated with upregulation of programmed death ligand-1 (22). Using a similar mouse model of skin graft transplantation, treatment with a mixture of rapamycin- and tacrolimus-loaded nanomicelles was shown to effectively target multiple immune cell subsets in the lymph node, with a prolonged allograft survival (23). Moreover, locally controlled and sustained release of corticosteroids using a biodegradable nanoparticle system after corneal transplantation prevents graft rejection in rats (24). In conclusion, these studies provide a comprehensive *in vitro* and *in vivo* evidence for the superiority of PLGA encapsulated immunomodulatory drugs over the soluble form and its potential in organ transplantation.

In summary, nanoparticles are used for the delivery of low dose immunosuppressive agents in conjunction with antigens to prevent specific immune responses. These studies mostly used nanotherapies based on the Food and Drug Administration-approved poly(lactic-co-glycolic acid) (PLGA) nanoparticles. While biodegradable, PLGA nanoparticles are large (≥100 nm in diameter), tend to aggregate, and are taken up by all phagocytic cells in a non-specific manner. The nanoparticle size is a critical factor for uptake and retention in the lymphoid secondary organs, since small nanoparticles (≤25 nm) are taken up more efficiently and retained for longer periods (25). Altogether, nanotherapeutic specific targeting of the APCs represents a promising strategy to inhibit the upstream steps of transplant rejection and to generate a durable donor-specific tolerant state.

## HDL-NPs as Nanocarriers for Drug Delivery to APCs in Transplantation

High-density lipoproteins are natural, small dynamic nanoparticles that have immuno protective function through macrophage targeting (26). They are being exploited in atherosclerosis, as a nanotherapeutic option (27) and are also used for targeting tumor-associated macrophages and as a cancer therapeutic tool (28, 29). Since HDL-NPs exhibit high specificity toward myeloid cells, they deliver immune modulatory drugs to APCs *in vivo* (30). Apolipoprotein A-I (apoA-I) is the main amphipathic lipoprotein associated with HDL-NPs and defines the size and shape of these nanoparticles (28, 31). HDL-NPs preferentially interact with receptors that are highly expressed by myeloid cells, including ATP-binding cassette receptor A1 and scavenger receptor type B-1 (32). As a result, HDL-NPs represent an attractive approach to *in vivo* target myeloid cells in transplant recipients. Their ability to incorporate therapeutic agents can be used to specifically deliver immunosuppressive drugs to the innate immune system and prevent the immune alloreactivity, thus promoting long-term allograft survival. Since the biodistribution of HDL-NPss is tightly dependent on their composition, the number of apoA-I molecules, their purity, and ratio relative to other nanoparticle components, such as phospholipids, need additional investigation for optimal results. Ultimately, HDL-NPs synthesis should be adapted to each disease to provide the best and most specific tissue and cell targeting tool (33).

## Nanoparticles for APCs Monitoring in Transplantation: Imaging Approaches

Besides their use as drug delivery carriers, nanoparticles can also be used to image a biological process. Pioneer approaches to imaging transplant rejection used radiolabeled anti-myosine antibody Fab fragments as a non-invasive detection of human cardiac transplant recipient rejection (34). Besides, magnetic resonance imaging (MRI) was used for repetitive imaging of transplanted hearts because it combines high spatial resolution with the ability to measure heart function while avoiding radiation exposure (35). Indeed, *in vivo* electrocardiographically gated MRI has been reported as a sensitive, non-invasive modality for the detection and the grading of cardiac transplant acute rejection, which correlates with the T2 relaxation times value (35). Even though gadolinium-based contrast agent play an important role in molecular and cellular imaging (36), most MRI cellular studies rely on the superior sensitivity of superparamagnetic or ultrasmall superparmagnetic iron oxide nanoparticles for imaging contrast. MRI-sensitive iron oxide approach exploits the phagocytic capacity of myeloid cells, specifically macrophages to monitor allograft rejection (37, 38).

More recently, nanoparticles were used to visualize macrophages *in vivo* and for assessing their absolute number, flux rate, and functional state in different tissues and models (39–41). Radiolabeled and dextran crosslinked nanoparticles have been used as a macrophage-specific imaging agent for positron emission tomography (39, 42, 43). Furthermore, magnetic nanoparticles could also be used as probes for MRI to examine the function of immune cells in humans. However, using MRI for *in vivo* cell quantitation in organs is often complicated and needs more concentrated magnetic materials than radiolabeled nanoparticles (44). PEGylated gold nanoparticles and other nanoparticle-based contrast agents have been used also for X-ray computed tomography (CT) (45). Differently from the other described imaging techniques, X-ray CT requires a high concentration of nanoparticles to follow the macrophage populations. Therefore, different nanoparticle platforms can be used in personalized clinical care to provide diagnostic and prognostic information as well as for quantifying the treatment efficacy of transplant patients (Figure 1B).

## Concluding Remarks and Outstanding Questions

The use of nanoparticles represents a promising therapeutic strategy to target APCs *in vivo* and negatively modulate the immune response in organ transplant recipients. Nanoparticles are capable to induce antigen-specific myeloid cells with suppressive function that promote regulatory T cells expansion (46). Therefore, the immunosuppressive effects of nanoparticles loaded with donor antigens are ultimately transplant- and patient-specific. In addition, assays that evaluate the robustness of this nanotherapeutic approach and potentially distinguish between tolerant and non-tolerant patients need to be optimized. This could be in part be monitored using gene expression profiling of the patient’s blood, urine, or transplant biopsy as previously reported. As the final clinical objective is to maintain graft function and intact host defenses, a patient-specific genetic tolerogenic signature could be used to determine the frequency and dose of the nanotherapeutic treatment of each patient.

Protocols using nanoparticles for imaging in transplantation need to be optimized for their clinical application as a non-invasive approach to characterize and monitor the allograft function. While some animal models are being developed that evaluate the efficacy of nanoparticles in organ transplantation, much work is yet to be done to translate the results from bench to bedside. In addition, the precise mechanisms of action and the long-term effects of nanoparticles have not been fully elucidated yet. Although drug-loaded nanoparticles have demonstrated lower toxicity than the soluble form, the potential long-term toxicity and side effects of nanoparticles are not fully known. Interestingly, drug-loaded nanoparticles could be used as a combination therapy with other induction therapy strategies, such as thymoglobulin and interleukin-2 (IL-2) receptor antibodies (47). In this respect, it is important to test whether combined approaches that use drug-loaded nanoparticles are optimized in a mechanism-independent fashion and to determine the potential synergistic effects. Collectively, the use of nanoparticles as a targeted delivery approach that modulates APCs *in vivo* represent an innovative therapeutic protocol to prevent undesirable immune responses and promote long-term organ acceptance in transplant recipients direct translation into the clinical practice.

## Author Contributions

All authors listed have made a substantial, direct, and intellectual contribution to the work and approved it for publication.

## Conflict of Interest Statement

The authors declare that the research was conducted in the absence of any commercial or financial relationships that could be construed as a potential conflict of interest.

## Abbreviations

APC, antigen-presenting cell; DC, dendritic cell; FDA, Food and Drug Administration; IFN, interferon; IL, interleukin; HDL, high-density lipoprotein; HDL-NPs, high-density lipoprotein nanoparticles; PET, positron emission tomography; PLGA, poly(lactic-co-glycolic acid).

## References

[1] Demir T Ozel L Gokce AM Ata P Kara M Eris C Cancer screening of renal transplant patients undergoing long-term immunosuppressive therapy. Transplant Proc (2015) 47:1413–7. 10.1016/j.transproceed.2015.04.073 26093731

[2] Kupeli E Ulubay G Dogrul I Birben O Seyfettin P Ozsancak Ugurlu A Long-term risk of pulmonary embolism in solid-organ transplant recipients. Exp Clin Transplant (2015) 13(Suppl 1):223–7. 10.6002/ect.mesot2014.P205 25894159

[3] Lien YH Top 10 things primary care physicians should know about maintenance immunosuppression for transplant recipients. Am J Med (2009) 129:568–72. 10.1016/j.amjmed.2015.11.034 26714210

[4] Gardiner KM Tett SE Staatz CE. Multinational evaluation of mycophenolic acid, tacrolimus, cyclosporin, sirolimus, and everolimus utilization. Ann Transplant (2016) 21:1–11. 10.12659/AOT.895664 26729299

[5] Auchincloss H No tolerance for depletion. Nat Med (2004) 10:21–3. 10.1038/nm0104-21 14702625

[6] Wu Z Bensinger SJ Zhang J Chen C Yuan X Huang X Homeostatic proliferation is a barrier to transplantation tolerance. Nat Med (2004) 10:87–92. 10.1038/nm965 14647496 PMC2839903

[7] Zecher D Van Rooijen N Rothstein DM Shlomchik WD Lakkis FG. An innate response to allogeneic nonself mediated by monocytes. J Immunol (2009) 183:7810–6. 10.4049/jimmunol.0902194 19923456

[8] Liu W Xiao X Demirci G Madsen J Li XC. Innate NK cells and macrophages recognize and reject allogeneic nonself in vivo via different mechanisms. J Immunol (2012) 188:2703–11. 10.4049/jimmunol.1102997 22327074 PMC3298083

[9] Oberbarnscheidt MH Zeng Q Li Q Dai H Williams AL Shlomchik WD Non-self recognition by monocytes initiates allograft rejection. J Clin Invest (2014) 124:3579–89. 10.1172/JCI74370 24983319 PMC4109551

[10] Conde P Rodriguez M Van Der Touw W Jimenez A Burns M Miller J DC-SIGN(+) macrophages control the induction of transplantation tolerance. Immunity (2015) 42:1143–58. 10.1016/j.immuni.2015.05.009 26070485 PMC4690204

[11] Kono H Rock KL. How dying cells alert the immune system to danger. Nat Rev Immunol (2008) 8:279–89. 10.1038/nri2215 18340345 PMC2763408

[12] Dai H Friday AJ Abou-Daya KI Williams AL Mortin-Toth S Nicotra ML Donor SIRPalpha polymorphism modulates the innate immune response to allogeneic grafts. Sci Immunol (2017) 2. 10.1126/sciimmunol.aam6202 28783664 PMC5653256

[13] Jose MD Ikezumi Y Van Rooijen N Atkins RC Chadban SJ. Macrophages act as effectors of tissue damage in acute renal allograft rejection. Transplantation (2003) 76:1015–22. 10.1097/01.TP.0000083507.67995.13 14557746

[14] Garcia MR Ledgerwood L Yang Y Xu J Lal G Burrell B Monocytic suppressive cells mediate cardiovascular transplantation tolerance in mice. J Clin Invest (2010) 120:2486–96. 10.1172/JCI41628 20551515 PMC2898596

[15] Swirski FK Wildgruber M Ueno T Figueiredo JL Panizzi P Iwamoto Y Myeloperoxidase-rich Ly-6C+ myeloid cells infiltrate allografts and contribute to an imaging signature of organ rejection in mice. J Clin Invest (2010) 120:2627–34. 10.1172/JCI42304 20577051 PMC2898607

[16] Azad AK Rajaram MV Schlesinger LS. Exploitation of the macrophage mannose receptor (CD206) in infectious disease diagnostics and therapeutics. J Cytol Mol Biol (2014) 1. 10.13188/2325-4653.1000003 24672807 PMC3963702

[17] Tel J Sittig SP Blom RA Cruz LJ Schreibelt G Figdor CG Targeting uptake receptors on human plasmacytoid dendritic cells triggers antigen cross-presentation and robust type I IFN secretion. J Immunol (2013) 191:5005–12. 10.4049/jimmunol.1300787 24127556

[18] Cruz LJ Rosalia RA Kleinovink JW Rueda F Lowik CW Ossendorp F. Targeting nanoparticles to CD40, DEC-205 or CD11c molecules on dendritic cells for efficient CD8(+) T cell response: a comparative study. J Control Release (2014) 192:209–18. 10.1016/j.jconrel.2014.07.040 25068703

[19] White KL Rades T Furneaux RH Tyler PC Hook S. Mannosylated liposomes as antigen delivery vehicles for targeting to dendritic cells. J Pharm Pharmacol (2006) 58:729–37. 10.1211/jpp.58.6.0003 16734974

[20] Zou W Cao G Xi Y Zhang N. New approach for local delivery of rapamycin by bioadhesive PLGA-carbopol nanoparticles. Drug Deliv (2009) 16:15–23. 10.1080/10717540802481307 19555304

[21] Haddadi A Elamanchili P Lavasanifar A Das S Shapiro J Samuel J. Delivery of rapamycin by PLGA nanoparticles enhances its suppressive activity on dendritic cells. J Biomed Mater Res A (2008) 84:885–98. 10.1002/jbm.a.31373 17647224

[22] Shirali AC Look M Du W Kassis E Stout-Delgado HW Fahmy TM Nanoparticle delivery of mycophenolic acid upregulates PD-L1 on dendritic cells to prolong murine allograft survival. Am J Transplant (2011) 11:2582–92. 10.1111/j.1600-6143.2011.03725.x 21883921

[23] Dane KY Nembrini C Tomei AA Eby JK O’neil CP Velluto D Nano-sized drug-loaded micelles deliver payload to lymph node immune cells and prolong allograft survival. J Control Release (2011) 156:154–60. 10.1016/j.jconrel.2011.08.009 21864593

[24] Pan Q Xu Q Boylan NJ Lamb NW Emmert DG Yang JC Corticosteroid-loaded biodegradable nanoparticles for prevention of corneal allograft rejection in rats. J Control Release (2015) 201:32–40. 10.1016/j.jconrel.2015.01.009 25576786 PMC6037178

[25] Reddy ST Van Der Vlies AJ Simeoni E Angeli V Randolph GJ O’neil CP Exploiting lymphatic transport and complement activation in nanoparticle vaccines. Nat Biotechnol (2007) 25:1159–64. 10.1038/nbt1332 17873867

[26] Duivenvoorden R Tang J Cormode DP Mieszawska AJ Izquierdo-Garcia D Ozcan C A statin-loaded reconstituted high-density lipoprotein nanoparticle inhibits atherosclerotic plaque inflammation. Nat Commun (2014) 5:3065. 10.1038/ncomms4065 24445279 PMC4001802

[27] Tang J Lobatto ME Hassing L Van Der Staay S Van Rijs SM Calcagno C Inhibiting macrophage proliferation suppresses atherosclerotic plaque inflammation. Sci Adv (2015) 1 10.1126/sciadv.1400223 PMC4539616 26295063

[28] McMahon KM Foit L Angeloni NL Giles FJ Gordon LI Thaxton CS. Synthetic high-density lipoprotein-like nanoparticles as cancer therapy. Cancer Treat Res (2015) 166:129–50. 10.1007/978-3-319-16555-4_6 25895867 PMC4418545

[29] Thaxton CS Rink JS Naha PC Cormode DP. Lipoproteins and lipoprotein mimetics for imaging and drug delivery. Adv Drug Deliv Rev (2016) 106(Pt A):116–31. 10.1016/j.addr.2016.04.020 27133387 PMC5086317

[30] Kuai R Li D Chen YE Moon JJ Schwendeman A. High-density lipoproteins: nature’s multifunctional nanoparticles. ACS Nano (2016) 10:3015–41. 10.1021/acsnano.5b07522 26889958 PMC4918468

[31] Camont L Chapman MJ Kontush A. Biological activities of HDL subpopulations and their relevance to cardiovascular disease. Trends Mol Med (2011) 17:594–603. 10.1016/j.molmed.2011.05.013 21839683

[32] Yang XP Amar MJ Vaisman B Bocharov AV Vishnyakova TG Freeman LA Scavenger receptor-BI is a receptor for lipoprotein(a). J Lipid Res (2013) 54:2450–7. 10.1194/jlr.M038877 23812625 PMC3735942

[33] Pirillo A Norata GD Catapano AL High-density lipoprotein subfractions – what the clinicians need to know. Cardiology (2013) 124:116–25. 10.1159/000346463 23428644

[34] Frist W Yasuda T Segall G Khaw BA Strauss HW Gold H Noninvasive detection of human cardiac transplant rejection with indium-111 antimyosin (Fab) imaging. Circulation (1987) 76:V81–5. 3311460

[35] Aherne T Tscholakoff D Finkbeiner W Sechtem U Derugin N Yee E Magnetic resonance imaging of cardiac transplants: the evaluation of rejection of cardiac allografts with and without immunosuppression. Circulation (1986) 74:145–56. 10.1161/01.CIR.74.1.145 3518982

[36] Aime S Barge A Cabella C Crich SG Gianolio E. Targeting cells with MR imaging probes based on paramagnetic Gd(III) chelates. Curr Pharm Biotechnol (2004) 5:509–18. 10.2174/1389201043376580 15579040

[37] Kanno S Wu YJ Lee PC Dodd SJ Williams M Griffith BP Macrophage accumulation associated with rat cardiac allograft rejection detected by magnetic resonance imaging with ultrasmall superparamagnetic iron oxide particles. Circulation (2001) 104:934–8. 10.1161/hc3401.093148 11514382

[38] Wu YL Ye Q Foley LM Hitchens TK Sato K Williams JB In situ labeling of immune cells with iron oxide particles: an approach to detect organ rejection by cellular MRI. Proc Natl Acad Sci U S A (2006) 103:1852–7. 10.1073/pnas.0507198103 16443687 PMC1413627

[39] Nahrendorf M Zhang H Hembrador S Panizzi P Sosnovik DE Aikawa E Nanoparticle PET-CT imaging of macrophages in inflammatory atherosclerosis. Circulation (2008) 117:379–87. 10.1161/CIRCULATIONAHA.107.741181 18158358 PMC2663426

[40] Weissleder R Nahrendorf M Pittet MJ. Imaging macrophages with nanoparticles. Nat Mater (2014) 13:125–38. 10.1038/nmat3780 24452356

[41] Perez-Medina C Tang J Abdel-Atti D Hogstad B Merad M Fisher EA PET imaging of tumor-associated macrophages with 89Zr-labeled high-density lipoprotein nanoparticles. J Nucl Med (2015) 56:1272–7. 10.2967/jnumed.115.158956 26112022 PMC4737475

[42] Keliher EJ Yoo J Nahrendorf M Lewis JS Marinelli B Newton A 89Zr-labeled dextran nanoparticles allow in vivo macrophage imaging. Bioconjug Chem (2011) 22:2383–9. 10.1021/bc200405d 22035047 PMC3244512

[43] Nahrendorf M Keliher E Marinelli B Leuschner F Robbins CS Gerszten RE Detection of macrophages in aortic aneurysms by nanoparticle positron emission tomography-computed tomography. Arterioscler Thromb Vasc Biol (2011) 31:750–7. 10.1161/ATVBAHA.110.221499 21252070 PMC3060293

[44] Gaglia JL Guimaraes AR Harisinghani M Turvey SE Jackson R Benoist C Noninvasive imaging of pancreatic islet inflammation in type 1A diabetes patients. J Clin Invest (2011) 121:442–5. 10.1172/JCI44339 21123946 PMC3007157

[45] Cormode DP Roessl E Thran A Skajaa T Gordon RE Schlomka JP Atherosclerotic plaque composition: analysis with multicolor CT and targeted gold nanoparticles. Radiology (2010) 256:774–82. 10.1148/radiol.10092473 20668118 PMC2923725

[46] Jhunjhunwala S Raimondi G Glowacki AJ Hall SJ Maskarinec D Thorne SH Bioinspired controlled release of CCL22 recruits regulatory T cells in vivo. Adv Mater (2012) 24:4735–8. 10.1002/adma.201202513 22821823 PMC3491880

[47] Hellemans R Bosmans JL Abramowicz D. Induction therapy for kidney transplant recipients: do we still need anti-IL2 receptor monoclonal antibodies? Am J Transplant (2017) 17:22–7. 10.1111/ajt.13884 27223882 PMC5215533

